# TopFed: TCGA tailored federated query processing and linking to LOD

**DOI:** 10.1186/2041-1480-5-47

**Published:** 2014-12-03

**Authors:** Muhammad Saleem, Shanmukha S Padmanabhuni, Axel-Cyrille Ngonga Ngomo, Aftab Iqbal, Jonas S Almeida, Stefan Decker, Helena F Deus

**Affiliations:** Universität Leipzig, IFI/AKSW, PO 100920, D-04009 Leipzig, Germany; Insight Centre for Data Analytics, National University of Ireland (NUIG), Galway, Ireland; Division Informatics, Department of Pathology, University of Alabama, Birmingham, USA; Foundation Medicine, Inc, Cambridge, MA 02141 USA

**Keywords:** Federated queries, SPARQL, TCGA, RDF

## Abstract

**Backgroud:**

The Cancer Genome Atlas (TCGA) is a multidisciplinary, multi-institutional effort to catalogue genetic mutations responsible for cancer using genome analysis techniques. One of the aims of this project is to create a comprehensive and open repository of cancer related molecular analysis, to be exploited by bioinformaticians towards advancing cancer knowledge. However, devising bioinformatics applications to analyse such large dataset is still challenging, as it often requires downloading large archives and parsing the relevant text files. Therefore, it is making it difficult to enable virtual data integration in order to collect the critical co-variates necessary for analysis.

**Methods:**

We address these issues by transforming the TCGA data into the Semantic Web standard Resource Description Format (RDF), link it to relevant datasets in the Linked Open Data (LOD) cloud and further propose an efficient data distribution strategy to host the resulting 20.4 billion triples data via several SPARQL endpoints. Having the TCGA data distributed across multiple SPARQL endpoints, we enable biomedical scientists to query and retrieve information from these SPARQL endpoints by proposing a TCGA tailored federated SPARQL query processing engine named TopFed.

**Results:**

We compare TopFed with a well established federation engine FedX in terms of source selection and query execution time by using 10 different federated SPARQL queries with varying requirements. Our evaluation results show that TopFed selects on average less than half of the sources (with 100% recall) with query execution time equal to one third to that of FedX.

**Conclusion:**

With TopFed, we aim to offer biomedical scientists a single-point-of-access through which distributed TCGA data can be accessed in unison. We believe the proposed system can greatly help researchers in the biomedical domain to carry out their research effectively with TCGA as the amount and diversity of data exceeds the ability of local resources to handle its retrieval and parsing.

## Background

The Cancer Genome Atlas [1] (TCGA) is an effort led by the National Cancer Institute to characterize and sequence more than 30 cancer types from 9000 patients at the molecular level. The goal is to analyse DNA for every participant to discover abnormalities present in a tumour sample that are peculiar to the oncogenic process and whether it affect progression and regression of the tumours. Each cancer type published by TCGA has three levels. Level 1 is raw data, level 2 is normalized data, and level 3 is processed data. The analytics are performed on the level 3 data, which is also of our interest for the work presented in this paper. TCGA is a valuable resource for hypothesis-driven translational research as all of its data results from direct experimental evidence. Analysis of such evidence within cancer research has led in recent years to clinically relevant findings in the genetic mark-ups of different cancer types and is at the forefront of a coordinated worldwide effort towards making more molecular results from cancer analyses publicly available [2].

Big data research initiatives such as the International Cancer Genomics Consortia [3], the 1000genomes [4] and the One Million Genomes project [5], the $10 Million Genome Prize [6], and the remarkable drop in the cost of genome sequencing [7] will soon mean that the current bioinformatics paradigm in which researchers download all the data, extract the interesting pieces and remove the rest, will no longer be feasible [8, 9]. The rapid development of advanced statistical methods for analysing cancer genomics [10–12] further emphasizes the need to enable smooth online data collection and aggregation. As pointed out in [13], “Large-scale genome characterization efforts involve the generation and interpretation of data at an unprecedented scale that has brought into sharp focus the need for improved information technology infrastructure and new computational tools to render the data suitable for meaningful analysis”. A scalable and robust solution is therefore a critical requirement, whereby researchers can obtain a subset of big data they are interested in by executing a query using a particular service.

In addition to the large semi-structured experimental results available through TCGA and related projects, there is a significant number of unstructured and structured biomedical datasets available on the Web. Most of these datasets are critical towards annotating and integrating the experimental results. Remote query processing and virtual data integration, i.e., transparent on-the-fly-view creation for the end user, can provide a scalable solution to both challenges. Due to the majority of TCGA data being available in text files (in tabular format), it is difficult to query the contents of a particular file or to enable virtual data integration. In this paper, we have addressed above problems by applying Semantic Web technologies and federated query processing. Semi-structured level 3 TCGA data were converted into Semantic Web standard format RDF such that it could be queried and publicly accessed via SPARQL endpoints. This choice of technology complies with the W3C recommendation of integrating distributed and heterogeneous data sources. There are currently a large number of applications supporting SPARQL and RDF, both academic and commercial, and both SwissProt [14] and EBI [15] have made their databases available as SPARQL endpoints.

In order to address the scalability issue while dealing with big data, we propose an efficient data distribution strategy and a TCGA tailored federated query engine (named TopFed) that leverages the data distribution along with the structure of triple pattern joins in a query for smart source selection. The logistics of the proposed solution will be assessed by comparison with a well established federation engine FedX [16].

### Motivation

Before TCGA, most cancer genomics studies have focused on only one type of data or one cancer histology. The Cancer Genome Atlas project changes that paradigm by making available to oncologists and biomedical scientists a comprehensive compilation of raw and processed data files on over 30 different cancer histologies and at several levels of “Genomics” (e.g. SNP, protein expression, exon expression, sequences, methylation, etc.). Since 2006, when the Cancer Genome Atlas first became available, multiple studies were devised to exploit its data. Nevertheless, a means to easily exploit this “cancer atlas” like one would exploit an atlas of planet Earth, does not yet exist. Part of the challenge is caused by a need to represent, organize and structure the 28.3 TB of data [17] available to the public in a way that can be easily queried by computational/statistics tools. Further complicating this task has been the growth of TCGA data. Some institutions have access to the computational resources necessary to provide a TCGA-synchronized and query-able interface suitable to address the most complex questions such as comparing methylation across cancer histologies or correlating exon expression results with methylation patterns regardless of cancer histology. One institution providing a tool and query language to exploit this data is Memorial Sloan Kettering through its cBio portal [18]. However, the data must first be constrained to the type of cancer before it can be exploited from a biological/molecular stand point. A second challenge is caused by the applications of the data - not all data are useful for all cancer researchers. Some researchers focus on a particular type of data, or a particular cancer histology, and therefore have little or no interest in hosting the entire Cancer Genome Atlas in a structured, query-able form.

The aim behind the work presented in this paper was to develop the computational concepts - and devise a prototype - that enable the exposure of TCGA as a distributed, semantically aware API (application programming interface). Although the data can be freely downloaded and analyzed by anyone with a sufficiently powerful computer, the computational tools available nowadays do not enable exploring this “atlas” without significant effort involved in selecting and downloading the data, mapping it to genomic coordinates and easily navigating to the sections of the genome that are relevant for understanding cancer. For example, zooming into genomic regions known as “Cancer Hotspots” or into the genomic coordinates where oncogenes and tumour suppressors are encoded, requires a combination of efforts including: 1) downloading the data; 2) parsing the text files for relevant results; and 3) mapping each file to the same set of genomic coordinates. On the other hand, fast, easy to use and integrated access to the big data such as TCGA requires: 1) Representing data in a format (e.g. RDF) amenable to integrated search; 2) logically connect all data; 3) distributing data across multiple locations (load balancing); and 4) supporting linking and federated querying (collecting data from more than one location using a single query) with external data sources.

TopFed is devised to address these requirements. Whereas requirements 1 and 2 are addressed using RDF and class level connectivity (see section TCGA Data Work flow), addressing requirements 3 and 4 relies on techniques that make the best use of the architecture of the Web to enable both redundancy when resources are down and sharing the load of hosting this data across multiple locations. As a proof of concept, TopFed links different portions of the Cancer Genome Atlas across two institutions, one at Insight Centre for Data Analytics at NUIG in Ireland and other at the University of Alabama at Birmingham in United States. TopFed is devised as a federation query engine that enables selection of the appropriate endpoints necessary to address an incoming query as well as optimization of the services discovery based on metadata about each endpoint. TopFed accepts queries in SPARQL, the same universal, standardized query language as each of the endpoints connected to it, making its functionality straightforward. For example, if someone is looking to query only one cancer histology, they can direct their queries at the endpoint hosting that data. However, if someone wants to exploit and compare multiple cancer histologies, the query can be pointed at TopFed, which automates and optimizes the task of discovering endpoints that contain the data necessary to address the question. To illustrate a typical use case, we exemplify a genomic region query enabled by TopFed.

### Biological query example

**Figure 1:**
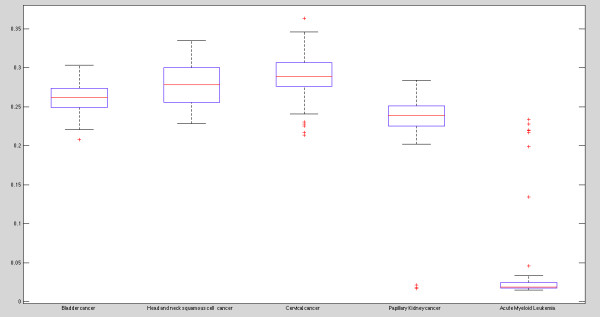
**Biological query results.** We used TopFed to search for the methylation status of the KRAS gene (chr12:25386768-25403863) across five cancer histologies (hosted by five SPARQL endpoints) and created a box plot comparing the methylation values. The corresponding SPARQL query to retrieve the required methylation values is given in Listing 1.

This example makes use of the KRAS gene, a gene that is commonly mutated and constitutively active in many cancer types, leading the cell to replicate DNA and make copies of itself at a very fast pace. Genes with this type of behaviour in the cell are commonly called oncogenes. When mutated, these genes become constitutive active, thus having the potential to cause normal cells to become cancerous. Imagine that for five different cancer histologies, we used TopFed to search for the methylation status of the KRAS gene (chr12:25386768-25403863), and created a box plot comparing the values, shown in Figure 1. The query (given in Listing 1) executed on each of the five SPARQL endpoints^a^, resulting in five different samples.

#### Listing 1 Query to retrieve average methylation values for the KRAS gene and for all patient of a particular cancer type



This query returns the average methylation results for the KRAS gene of all patients in a particular cancer histology. The results show a clear distinction between solid tumours and hematopoetic cancers. This differential in the methylation values is not necessarily surprising results, given that blood cancers are known to be significantly different genetically from solid tumours. What is interesting and worth further exploring in these cases is the shape of the distribution: why Acute Myeloid Leukemia (AML) samples, a cancer of the myeloid of blood cells, appear to have high methylation, effectively creating a bi-modal distribution? Exploring the provenance of this data may provide a clue for that - one hypothesis is that these samples were incorrectly diagnosed as AML or it may be that these AML samples are indeed genetically different - and therefore should not be treated with the same therapies as the others. Since TopFed integrates both the clinical and genomic parameters, exploring these different hypothesis is as easy as returning to the query and retrieving the potentially relevant clinical parameters that could explain the difference. Exploring the same gene (KRAS) in another type of data (e.g. exon expression) could also help explain why these samples are different. Since TopFed is “aware” of which SPARQL endpoints store each data property, it will appropriately select the correct source for the data, thereby adding the extra parameters to the query sufficient to generate sufficiently robust hypothesis.

Further exploring these examples is beyond the scope of this manuscript - however, we encourage our readers to experiment themselves with their own hypothesis or with a different set of genes/genomic locations by changing the values for tcga:chromosome and tcga:position. We include an example of a query that could be used to retrieve the clinical parameters for the outlier patients (and compare with non-outlier patients) in Listing 2.

#### Listing 2 Query to retrieve average methylation values for the KRAS gene, along with clinical data, for all AML outlier patients. This query can be run at http://vmlion14.deri.ie/node45/8082/sparql



### Related work

The TCGA data has been widely used in the literature, but mostly in its raw form. Verhaakl et al. [19] use the gene expression results to describe a robust molecular classification of TCGA Glioblastoma Multiforme (GBM) into Proneural, Neural, Classical, and Mesenchymal subtypes and integrate multidimensional genomic data to establish patterns of somatic mutations and DNA copy number. Other notable contributions [20–24], including our own early analysis of DNA copy number variation in GBM [25] make use of the TCGA data for various important findings without, however, using more than one or possibly two types of molecular data. To facilitate integrated analysis over all cancer types, Deus et al. developed an infrastructure using Simple Sloppy Semantic Database (S3DB) management model to expose clinical, demographic and molecular data elements generated by TCGA as a SPARQL endpoint [26]. Robbins et al. [27] developed an engine to continuously index and annotate the TCGA data files using JavaScript in conjunction with RDF, and the SPARQL query language. However, both [26] and [27] provide only file level provenance annotations without providing structured access to actual contents contained in the files. Recently, Saleem et al. [28] presented a Linked Data version of the Cancer Genome Atlas Database for effective cancer treatment. This work demonstrates three use cases namely target cancer treatment, mechanism-based cancer treatment, and survival outcome, where the Linked Data approach of integrating TCGA data was used. More recently, a visualization of the integration of the Linked TCGA cancer data with PubMed publications is presented in [29, 30]. The main aim behind this work is to foster serendipity through big data RDFization, continuous integration, and visualization. GenomeSnip, a visual analytics platform, which facilitates the intuitive exploration of the human genome and displays the relationships between different genomic features, is presented in [31].

Advances in federated query processing methods over the Web of Data have enabled the application of federated solutions for datasets, such as those from genomics. Quilitz and Leser [32] propose DARQ, which makes use of service descriptions for relevant data source selection.

Langegger et al. in [33] propose a solution similar to DARQ using a mediator approach, which continuously monitors the SPARQL endpoints for any dataset changes and updates the service descriptions automatically. Umbrich et al. [34, 35] propose a Qtree-based index structure that summarizes the content of data source for query execution over the Web of Data. Schwarte et al. [16] propose FedX, an index-free query federation for the Web of Data.

SPLENDID [36] makes use of VOID descriptions along with SPARQL ASK queries to select the list of relevant sources for each triple pattern. Both FedX and SPLENDID are able to handle more expressive queries as compared to previous contributions.

Other optimization techniques have also been attempted. Li and Heflin [37] built a tree structure that supported federated query processing over heterogeneous sources. Kaoudi et al. [38] propose a federated query technique on top of distributed hash tables (DHT). Ludwig and Tran [39] developed a mixed query engine that assumes some incomplete knowledge about the sources to select and discover new sources at run time. Acosta et al. [40] present ANAPSID, an adaptive query engine that adapts query execution schedulers to SPARQL endpoints data availability and run-time conditions.

Avalanche [41] gathers endpoint dataset statistics and bandwidth availability on-the-fly before the query federation. Saleem et al. [42] presented DAW, a novel duplicate-aware federated query approach over the Web of Data. DAW makes use of the min-wise independent permutations [43] and compact data summaries to extend existing SPARQL query federation engines in order to achieve the same query recall values while querying less SPARQL endpoints. Finally, HiBISCuS [44] is an efficient hypergraph based source selection approach for SPARQL query federation over multiple SPARQL endpoints. A fine-grained evaluation of SPARQL endpoint federation systems is performed in [45].

All of the above SPARQL query federation approaches are more generic and usually over-estimate (explained in the Source Selection sub-section below) the set of sources capable for answering a query. This over-estimation can be expensive when data is large. In our case, the data in hand is also large and we need a TCGA optimized federation engine that selects close to optimal set of capable sources. To this end, we propose TopFed, a TCGA tailed federated engine that make use of the intelligent data distribution and join-aware source selection to minimise the source over-estimation and provide fast query results.

The main contributions of this paper are the following:

1. We have proposed a Linked Data version of TCGA that supports efficient data distribution and federated SPARQL queries to integrate data from multiple SPARQL endpoints efficiently by only sending remote queries.
2. We have published, to the best of our knowledge, the largest RDF dataset (20.4 billion triples) and linked it to various datasets in the LOD cloud to enable annotation and enhancement with public knowledge bases as well as virtual data integration.
3. We devised the basic architecture and logic rules governing TopFed, a smart federated query engine for virtual integration of the TCGA data from multiple SPARQL endpoints that comply with the TCGA organizational schema. Further, we provide easy to use utilities [46] in order to refine and transform TCGA raw text files into RDF.
4. We evaluate our approach against FedX using 10 different SPARQL queries and show that our source selection algorithm, on average, selects less than half sources compared to FedX (with 100% recall). Also, our average query processing time is one third in comparison to FedX.

The remaining part of this paper is organized as follows: we present our methodology to refine, RDFize and link the TCGA data to LOD datasets in detail. Subsequently, we present a thorough evaluation of our approach against state of the art approaches. We finally conclude the paper with a discussion of our findings and an overview of future work.

## Methods

### Transforming TCGA data to RDF

**Figure 2:**
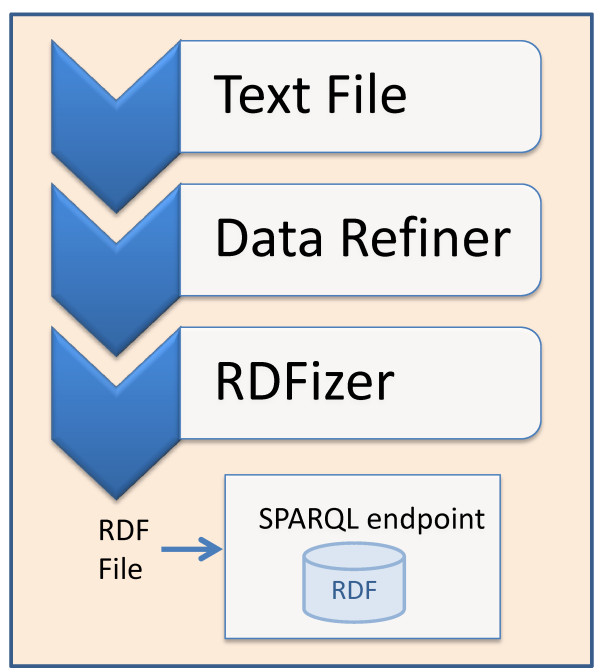
**TCGA text to RDF conversion process.** Given a text file, first it is refined by the Data Refiner. The refine file is then converted into RDF (N3 notation) by the RDFizer. Finally, the RDF file is uploaded into a SPARQL endpoint.

**Figure 3:**
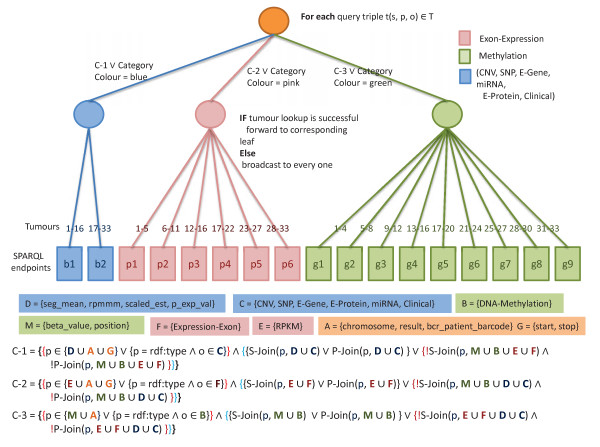
**TCGA data distribution/load balancing and source selection.** The proposed data distribution and source selection diagram for hosting the complete Linked TCGA data.

The process of transforming TCGA data into RDF^b^ is shown in Figure 2. Given a TCGA text file, the first processing step is carried out by the Data Refiner, which selects the specific fields [47] necessary for traditional molecular analysis algorithms. This step is necessary to minimize the size of the resulting RDF according to what we expect will be the most useful results. It is important to note that the above required fields for different types of results may not be directly accessible through raw text files. To this end, our Data Refiner makes use of the annotations files [48] for the required fields lookup. For example, methlylation annotation files are used to obtain chromosome and position values using Probe_Name lookup. Finally, the refined text file is sent to the RDFizer, which generates the resulting RDF dump in N3 format [49]. Our choice of N3 was due of its efficient space consumption. The generated RDF dumps^c^ are then uploaded to various SPARQL endpoints according to the distribution rules shown in Figure 3.

**Figure 4:**
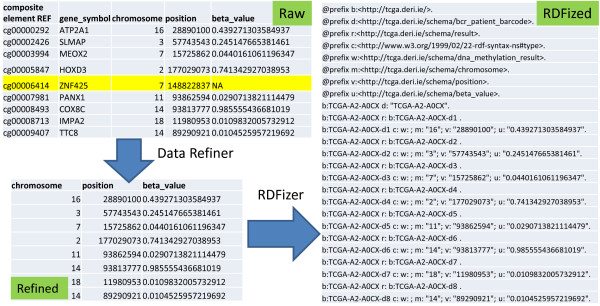
**Text to RDF conversion process example.** An example showing the refinement and RDFication of the TCGA file.

An example of the above RDFication process is shown in Figure 4, where part of raw methylation result of patient TCGA-A2-A0CX is provided as input to the Data Refiner. The Data Refiner selects *chrome, position*, and *beta_value* out of the five available columns. The selected columns are commonly used for traditional molecular analysis algorithms targeting methylation data. It is important to note that Data Refiner also skipped the yellow highlighted line because *beta_value* is not available for that specific methylation result. The refined text file is then passed to RDFizer that generates the RDF dump (N3 format). The values d1...d8 show DNA methylation results from 1 to 8. The use of this information is further explained in the Source Selection sub-section.

The accuracy of the text to RDF conversion is 100% (to the best of our understanding) since our Data Refiner selects a predefined set of fields for different types of results. Further, it skips specific field values (such as *NA, Null, Unknown, Not Reported etc.*) during RDFication process as shown in the above example. Currently, we have RDFized 27 cancer tumours and the statistics are shown in Table 1. We will RDFize new TCGA data once it is available through the TCGA data portal.

**Table 1:** **Statistics for 27 tumours sorted by number of triples**

Tumour type	Raw(GB)	Refined(GB)	RDF(GB)	Triples(Million)
Lymphoid Neoplasm Diffuse Large	0.37	0.20	0.83	35
B-cell Lymphoma (DLBC)				
Cutaneous melanoma (UCS)	1.2	0.64	2.6	113
Glioblastoma multiforme (GBM)	2.3	0.77	2.8	132
Esophageal carcinoma (ESCA)	1.5	0.88	3.4	149
Adrenocortical carcinoma (ACC)	1.6	0.90	3.6	158
Pancreatic adenocarcinoma (PAAD)	2.6	1.1	4.5	200
Kidney Chromophobe (KICH)	3.7	1.4	5.3	242
Sarcoma (SARC)	3.8	1.5	5.9	267
Cervical (CESC)	8.75	2.44	8.86	400.19
Ovarian serous cystadenocarcinoma (OV)	8.2	2.4	8.7	410
Rectal adenocarcinoma (READ)	8.07	2.25	9.04	413.31
Papillary Kidney (KIRP)	10.40	2.90	10.4	469.65
Stomach adenocarcinoma (STAD)	5.5	2.9	12	529
Liver hepatocellular carcinoma (LIHC)	8.2	3.1	12	550
Bladder cancer (BLCA)	12.16	3.39	12.3	556.38
Acute Myeloid Leukemia (LAML)	14.85	4.14	15.1	684.05
Lower Grade Glioma (LGG)	17.08	4.76	17.1	778.82
Prostate adenocarcinoma (PRAD)	18.05	5.03	18.1	821.01
Lung squamous carcinoma (LUSC)	20.63	5.75	20.5	927.08
Cutaneous melanoma (SKCM)	23.22	6.47	23.2	1050.94
Uterine Corpus Endometrial Carcinoma (UCEC)	13	5.98	24.2	1070
Colon adenocarcinoma (COAD)	18	6.64	26	1175
v Head and neck squamous cell(HNSC)	27.6	7.69	27.5	1245.37
Lung adenocarcinoma (LUAD)	23	9.1	36	1611
Kidney renal clear cell carcinoma (KIRC)	24	9.4	37	1658
Thyroid carcinoma (THCA)	26	10.1	40	1796
Breast invasive carcinoma (BRCA)	45	17	65	2959

A total of 20.4 Billion triples.

### Linking TCGA to the LOD cloud

One of the design principles of Linked Data [50] is the provision of links to other data sources. Adding links from TCGA to other knowledge bases is particularly crucial to ensure that the information already contained in other data sources can be easily (1) merged with the new TCGA data as well as (2) queried in combination with the TCGA data by means of federated SPARQL queries^d^. Moreover, links can facilitate other tasks such as cross-ontology question answering, data integration and data analytics. Yet, the sheer size of bio-medical knowledge base available on the LOD cloud and of the TCGA knowledge base itself makes it impossible to use manual linking to provide such cross knowledge-base links from TCGA to other data sources. We thus made use of the LIMES framework [51] for discovering links between TCGA and other knowledge bases. LIMES is a framework for link discovery that provides time-efficient implementations of several string and numeric similarity and distance measures. The framework provides both means to define link specifications explicitly and machine-learning algorithms for finding link specifications in an unsupervised and supervised fashion. Given that genes and chromosomes have dedicated IDs that are used across several biomedical knowledge bases, we used LIMES exactMatch measure for linking. We focused on linking patient data and lookup data with knowledge bases that describe genes and chromosomes. In particular, we linked TCGA to HGNC [52], OMIM [53] and Homologene [54]. Tables 2 and 3 provide an excerpt of the links generated for the TCGA dataset, while Listing 3 provides an excerpt of the specifications used for linking. The linking tasks were carried out on one kernel of a 2.3GHz i7 processor with 4GB RAM. Given that we used exact matches, we ensured that our link discovery achieves a precision of 100%. The recall of the linking process is tedious to assess as it would require assessing millions of links manually.

**Table 2:** **Excerpt of the links for the lookup files of TCGA**

Source	Target	Class	# links	Runtime (ms)
DNA27	HGNC	Genes	23,181	154
DNA27	Homologene	Genes	27,654	193
DNA27	OMIM	Genes	15,171	158
DNA450	Homologene	Genes	489,643	5,710
DNA450	OMIM	Genes	212,284	429
DNA27	HGNC	Chromosomes	108,662	96
DNA27	OMIM	Chromosomes	16,039,535	8,055

The source column shows the name of the look-up file that was linked to the target dataset named in the second column. The class column shows the type of resources that were linked. The fourth column shows the number of links that were generated while the runtime column shows the time required by LIMES to carry out the linking process in ms.

**Table 3:** **Excerpt of the links for the methylation results of a single patient**

Source	Target	Class	# links	Runtime (ms)
Methylation	HGNC	Chromosomes	97,530	205
Methylation	OMIM	Chromosomes	14,407,269	6,095
Gene expression	HGNC	Chromosomes	86,052	80
Gene expression	OMIM	Chromosomes	12,535,829	4,679

The source column shows the name of the patient file that was linked to the target dataset named in the second column. The class column shows the type of resources that were linked. The fourth column shows the number of links that were generated while the runtime column shows the time required by LIMES to carry out the linking process in ms.

#### Listing 3 Excerpt of the LIMES link specification for linking TCGA and Homologene



### TCGA data workflow and schema

**Figure 5:**
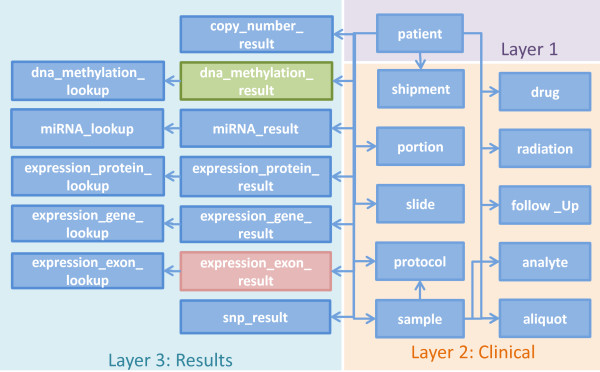
**TCGA class diagram of RDFized results.** Each level 3 data is further divided into three layers where: layer 1 contains patient data, layer 2 consists of clinical information and layer 3 contain results for different samples of a patient.

**Figure 6:**
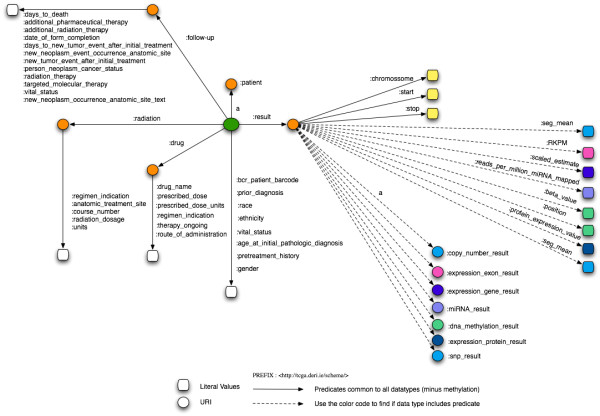
**Linked TCGA schema diagram.** The schema diagram of the Linked TCGA, useful for formulating SPARQL queries.

To devise a fast, big data driven query federation engine, we started by exploiting how the various files and types of data in TCGA are interconnected. To date, 23054 raw data files from 28 cancer tumours have been collected, summing up to a total of 28.3 TB of data [55]. For each level 3 data, we have identified three different types, i.e., we RDFized level 3 data for each cancer type and further define 3 data types for each of the level 3 tumours data of data. The resulting data are organized as a three layer architecture where layer 1 contains patient data, layer 2 consists of clinical information and layer 3 contain results for different samples of a patient. Each type of data is assigned to a different class in the RDFized version as depicted in Figure 5. For each patient, tumour and blood/normal tissue samples are collected and divided into different portions upon which different protocols such as DNA, RNA and so on, are applied to extract the analytes for the analysis of the sample. The extracted analytes are distributed across plates. All these plates containing patients tumour and normal samples are shipped to Genome Characterization Centres (GCCs) and Genome Sequencing Centres (GSCs) that produce different data type results which are shown in layer 3 (cf. Figure 5). The schema of the corresponding Linked TCGA is shown in Figure 6. We have included only important properties from clinical data (e.g., drug, follow-up, radiation etc.) as the complete list of properties is around 300. This diagram is useful to understand the connectivity between the Linked TCGA data and to formulate SPARQL queries.

### Data distribution and load balancing

**Figure 7:**
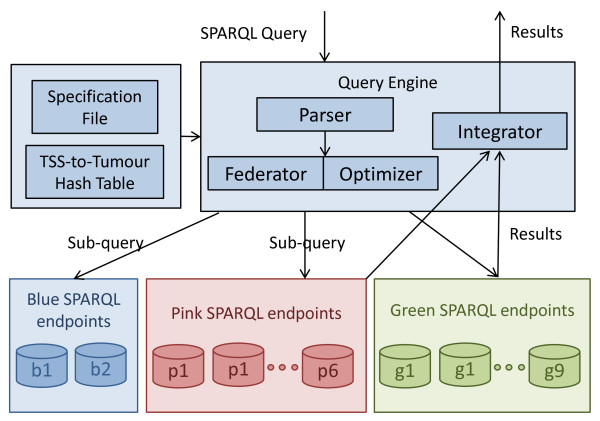
**TopFed federated query processing model.** TCGA tailored federated query processing diagram, showing system components.

A key property of the federation method described here is the efficient distribution of the data among SPARQL endpoints to enable access to around 20 billion resulting triples in a virtual integrated manner, i.e., the required data are transparently collected from different SPARQL endpoints. Proper load balancing among SPARQL endpoints is also ensured to reduce the query execution time. To this end, we have divided each tumour data into three categories, each of which is assigned a different colour – blue, pink and green – as shown in Figures 3 and 7. The green category contains only methylation results, pink contains expression exon results and all other data are grouped in the blue category. The ratio of the sizes is 1:3:4 for blue, pink, and green respectively.

In order to achieve proper load balancing, if we allocate one SPARQL endpoint to the blue category data (smallest) then we must assign three SPARQL endpoints to pink and four SPARQL endpoints to the green category data. We propose 17 SPARQL endpoints to be assigned for the complete TCGA level 3 data (around 33 tumours expected) distribution as shown in Figure 3. We assigned two SPARQL endpoints for blue, six endpoints for pink and nine endpoints for green category data.

Data are also balanced across each of the coloured category SPARQL endpoints according to cancer type (tumour). For example, in blue category, tumours1-16 are stored in the first blue SPARQL endpoint and the remaining tumours (17-33) are stored in the second blue SPARQL endpoint. It is important to note that we have RDFized 27 tumours while in our data distribution diagram we show 33 tumours. This is because we are expecting around 33 cancer tumours [56] data to be made available by the TCGA data portal in the future. To achieve a similar size-oriented division, each of the SPARQL endpoints in the pink category contains either five or six tumours data as shown in Figure 3 and each of the first six SPARQL endpoints in the green category contain data for four tumours and each of the remaining three SPARQL endpoints contain three tumours data. Each of the three categories is used to create a conditional statement (labelled C-1, C-2, and C-3 given in Listing 4), used by the federated engine for source selection. For source selection, the predicates sets shown in Figure 3 (D, C, B, M, F, E, A and G) are also relevant. We further explain the decision model in Source Selection sub-section.

#### Listing 4 Conditions for colour category selection



### TopFed federated query processing approach

Before going into the details of our federated query processing model shown in Figure 7, we first briefly explain TopFed’s index which comprise of an N3 specification file and a Tissue Source Site to Tumour (TSS-to-Tumour) hash table. The N3 specification file, shown in Listing 5, is devised based on the data distribution described in previous section. It contains metadata relevant for data distribution across SPARQL endpoints. For each SPARQL endpoint, its colour category, endpoint url, and the list of tumours data stored therein are specified. Moreover, the specification file also contains the various sets of predicates. In addition, we also create a Tissue Source Site to Tumour (TSS-to-Tumour [57]) hash table that contains key value pairs for TSS to tumour name. The TSS is the location identifier from where the results of the different tissues are obtained. This hash table was formed using “File_Sample_Map” files (containing file to patient barcode entries) provided as meta data, with every TCGA archive download via its Data Matrix portal^e^. This meta file provides a list of patient barcodes belonging to a particular cancer tumour. We extract the TSS part of patient barcode^f^ and use this along with tumour name as a hash entry. Both N3 specification file and TSS-to-Tumour hash table are used by our federated query processor for efficient relevant data source (SPARQL endpoints) selection, which is explained in the next sub-section.

#### Listing 5 Part of the N3 specification file



Given a SPARQL query, it is first parsed and then sent to the federator that makes use of the N3 specification file along with the TSS-to-Tumour hash table, in order to find the relevant sources for each of the triple pattern using Algorithm 1. The optimizer makes use of the source selection to generate an optimized sub-query execution plan. The optimized sub-queries are then forwarded to the relevant SPARQL endpoints. The results of each sub-query execution are integrated and the final query result set is generated.

### Source selection

The goal of the source selection is to find the optimal list of relevant sources (i.e., SPARQL endpoints) against individual query triple pattern. According to the distribution of Figure 3, if we can infer the category colour and tumour number for a triple pattern then we only need to query a single endpoint for that triple pattern. For example, starting from the root node of Figure 3, we can go to the second level of the tree by knowing the category colour (blue, pink, and green). Further, at second level, if we know the tumour number then we can reach to a single SPARQL endpoint to query. For each query triple pattern, our source selection algorithm tries to get such information using the specification file and type (star, path) of the join between the query triple patterns.

A star join between two triple patterns is formed if both of the triple patterns share the same subject. Consider the query given in Listing 6: the first two triple patterns form a star join and the last four triple patterns form a second star join. A path join between two triple patterns is formed if object of the first triple pattern is used as subject of the second triple pattern. For example, the second triple pattern form a path join with the third triple pattern in the query shown in Listing 6. Moreover, every TCGA patient is uniquely identified by its barcode of the format <TCGA-TSS-PatientNo>. For example, the patient barcode used in the first triple pattern of the Listing 6 query has a TSS identifier 18 and patient number 3406. This means we can infer tumour name/number from patient barcode using the TSS to tumour hash table.

#### Listing 6 TCGA query with bound predicate





As discussed in the Data distribution section, we have categorized all SPARQL endpoints into three different category colours named blue, pink, and green. Our source selection algorithm (cf. Algorithm 1) requires the set of SPARQL endpoints in each of the colour category and stores three different sets named *D*_*blue*_,*D*_*pink*_, and *D*_*green*_. Moreover, it requires the tumour number *tumourNo*, which can be null and is obtained from the query as follow: if a triple pattern with predicate tcga:bcr_patient_barcode and bound object containing the patient barcode form a star join with a triple pattern having predicate tcga:result, then by using the patient barcode value specified in the former triple pattern can be used to get the required tumour number using TSS-to-Tumour hash table. Our source selection algorithm runs for each basic graph pattern (BGP [58]) and for each individual triple pattern of BGP as follow.

If subject of the triple pattern is bound then we can get both the category colour and tumour name from the subject URI. The format of the TCGA URI is http://tcga.deri.ie/Patient_barcode-ResultType >. The tumour name can be obtained from Patient_Barcode and the category colour can be inferred from ResultType. For example, if the first character is *e* (shortcut for exon-expression), then it belongs to the pink category. However, if the first character is *d* (shortcut for dna-methylation), then it belongs to the green category and all other characters belong to the blue category. Consider the query given in Listing 7: the tumour name can be obtained using hash table lookup for TSS 18 and the colour category is pink.

#### Listing 7 TCGA query with bound subject



Source selection for a triple pattern with only bound predicate is more challenging. We have divided various predicates and classes of the TCGA data into different sets that are shown in Listing 8. Set *D* contains all the predicates that uniquely identify the blue category and set *C* contains a list of classes specific to it. The sets *B* and *M* uniquely identify the methylation, i.e., the green category while sets *F* and *E* are for the pink category. Sets *A* and *G* contain predicates that can be found in more than one colour category. Starting from the root of the source selection tree, if the condition *C-1* given in Listing 4 holds then all of the sources in blue category are relevant for that triple pattern. This means that if predicate *p* of the triple pattern is set member of {D ∪ A ∪ G} or it is equal to rdf:type and the object *o* belongs to set *C* and either the star or path join between *p* and {D ∪ C} is true or the star and path join of *p* with {M ∪ B ∪ E ∪ F} is false, then all of the sources in the blue category are relevant.

#### Listing 8 Predicate and class sets



Consider the third triple pattern of the query given in Listing 6: the predicate chromosome is set member of *A*, which means this predicate can be found in all of the endpoints. However, chromosome has a star join with seq_mean, which is unique for the blue category sources. Therefore, instead of selecting all of the sources (overestimated as in FedX, SPLENDID etc.), TopFed will only select *D*_*blue*_ as relevant sources that can be further filtered, provided that the tumourNo given as input to Algorithm 1 is not null. Similarly, *C-2* holds for *D*_*pink*_ and *C-3* holds for *D*_*green*_ relevant source selection. It is important to note that more than one condition (C-1, C-2, C-3) can be true for a triple pattern, therefore we check each of the three conditions individually and make a union of the sources as given in line 24 of Algorithm 1. Further, if none of the condition is true then we need to query the blue category sources because we did not list many of the blue category predicates as they are numerous.

For a triple pattern with bound object, we send SPARQL ASK queries including the triple pattern to all of the sources and select sources that pass the test. This is similar to the source selection technique used in FedX for all the triple patterns. Along with Algorithm 1, Figure 3 also provides a visual demonstration of our triple pattern-wise source selection.

As an example, consider the query of Listing 6 and the data distribution given in Figure 3. TopFed selects one source for the first triple pattern because we can obtain tumour number from the given patient barcode and this triple pattern only passes *C-1*. FedX selects three sources since every patient data can be found in each of the three colour categories exactly at one SPARQL endpoint. For the second triple pattern, TopFed again selects only one source because *C-1* only holds. However, FedX selects all of the 17 sources as predicate tcga:result can be found in all of the endpoints. For each of the remaining triple patterns (3 to 6), TopFex selects only one source as tcga:seq_mean is unique for the blue category endpoints and the others triple patterns (3 to 5) has star join with it. We have only two endpoints in blue category, which is filtered to one using the tumour number given in triple pattern 1. FedX selects all of the 17 sources for tcga:chromosome, eight sources each for tcga:start, tcga:stop, and two sources for last triple pattern. In total, TopFed selects only six sources while FedX selects 52 to answer this query. Additionally, FedX also needs to send 102 (6*17) SPARQL ASK queries. We want to emphasize that we have replaced only source selection algorithm of FedX. The join order optimization and the join implementation remains the same.

## Results and discussion

### Evaluation

The goal of this evaluation is to support the claim that TopFed selects a significantly smaller number of sources for the same recall as FedX, thus achieving a good query execution performance for large datasets. We compare TopFed with the state-of-the-art approach for query federation (FedX) both in terms of the total number of sources selected and the execution time to achieve a 100% recall, using 10 TCGA benchmark SPARQL queries^g^ of different shapes (i.e. star, path, and hybrid). A textual description of all the benchmark queries is given in Table 4. FedX has been shown previously [36, 45] to be the fastest and more precise SPARQL federated query engine (to the best of our knowledge). Therefore, we evaluate TopFed’s query performance by comparing it with FedX.

**Table 4:** **Benchmark queries descriptions**

Query	Description
Q1	Get the chromosome, start, stop and mean copy number values of the patient TCGA-18-4721 for genome locations 554268 to 5994290
Q2	Get the chromosome, start, stop and mean exon-expression values of all the TCGA patients
Q3	Get the chromosome, position and mean methylation values of all the TCGA patients
Q4	Get the chromosome, start and stop values of the TCGA patient TCGA-C4-A0F6
Q5	Get the chromosome, start, stop values of all the TCGA patients
Q6	Get the chromosome, start, stop and miRNA values of the 20th record of TCGA patient TCGA-AB-2821
Q7	Get the chromosome, start and stop values of the TCGA patient TCGA-AB-2823 for mean sequence value of 0.0839
Q8	Get the chromosome, start, stop, mean protein expression and mean exon-expression values of the TCGA patient TCGA-18-3410
Q9	Get the chromosome, mean gene expression and mean methylation values of the TCGA patient TCGA-C5-A1BF
Q10	Get the chromosome, mean gene expression, mean exon expression and mean methylation values of all the TCGA patients

The corresponding SPARQL queries can be downloaded from http://goo.gl/UxUEXk.

#### TCGA benchmark setup

TCGA benchmark data consists of genomic results from 25 patients randomly selected from ten different tumour types and distributed across ten local SPARQL endpoints with the specifications given in Table 5. Furthermore, the benchmark N3 specification^h^ file (used in the current experiments) assigns two, three, five SPARQL endpoints to the blue, pink, and green categories respectively.

**Table 5:** **Benchmark SPARQL endpoints specifications**

SPARQL endpoint	CPU	RAM	Hard disk
virtuoso-blue1	2.2 GHz, i3	4 GB	300 GB
virtuoso-blue2	2.6 GHz, i5	4 GB	150 GB
virtuoso-pink1	2.53 GHz, i5	4 GB	300 GB
virtuoso-pink2	2.3 GHz, i5	4 GB	500 GB
virtuoso-pink3	2.53 GHz, i5	4 GB	300 GB
virtuoso-green1	2.9 GHz, i7	16 GB	256 GB SSD
virtuoso-green2	2.9 GHz, i7	8 GB	450 GB
virtuoso-green3	2.6 GHz, i5	8 GB	400 GB
virtuoso-green4	2.6 GHz, i5	8 GB	400 GB
virtuoso-green5	2.9 GHz, i7	16 GB	500 GB

We have selected ten SPARQL queries based on expert opinion reflecting typical requests on TCGA data. Further, we have categorized our benchmark queries into four different quadrants as shown in Table 6. A single colour query collects results from SPARQL endpoints listed in one of the three colour categories. A cross-colour query targets more than one colour category results. A hybrid query contains both star and path joins between various triple patterns. Moreover, we can also obtain the tumour number (to be used as input to Algorithm 1) from all of the hybrid queries. All of the benchmark data, including benchmark queries, can be found at the project website.

**Table 6:** **Benchmark queries distribution**

	Single Colour	Cross-Colour
Star	2	2
Hybrid (star + path)	2	4

#### Experimental results

In order to show the effects of source selection on performance (runtime + recall of sources selected), the number of sources selected for each triple pattern of the query are added (equation 1). Let *m*_*i*_ equal the number of sources capable of answering a triple pattern *t*_*i*_ and *S* is the total number of available sources (10 in our benchmark). Then, for a query *q* with triple patterns { *t*_1_, *t*_2_, …, *t*_*n*_}, the total number of sources selected (triple pattern-wise sources selected) is given in equation 1.
1

**Figure 8:**
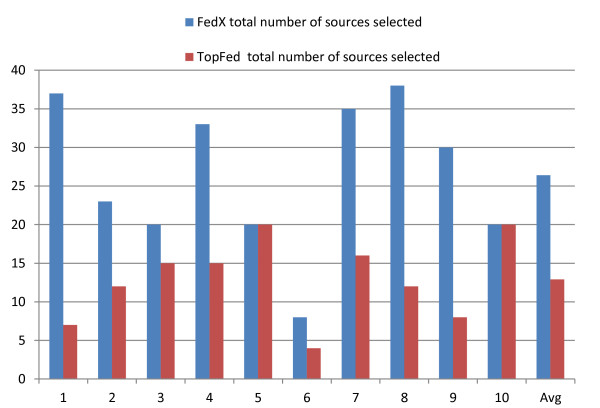
**Efficient source selection.** Comparison of the TopFed and FedX source selection in terms of the total number of triple pattern-wise sources selected. Y-axis shows the total triple pattern-wise sources selected for each of the benchmark query given in X-axis.

The source selection results are shown in Figure 8. Overall, our source selection algorithm selects on average less than half of the sources selected by FedX. This is due to the possible overestimation of the sources by FedX while using SPARQL ASK queries for relevant source selection [16]. For example, any data source will likely match a triple pattern (?s, rdf:type, ?o). However, the same sources might not lead to any results at all once the actual mappings for ?s and ?o are included in a join evaluation. On the contrary, our source selection algorithm was designed to resolve the join types between query triple patterns specifically to avoid such overestimation (which can later greatly increase the query processing time as reflected in Table 7). Only in queries 5 and 10, TopFed selected sources are equal to FedX. The explanation for this can be found in the amount of useful information available in each query - both query 5 and query 10 are generic queries from which a tumour or a performance-improving colour category cannot be derived, because all logic conditions are exactly satisfied. Overall, TopFed selects the optimal (the actual required sources) number of sources with 100% recall for all of the benchmark queries.

**Table 7:** **Comparison of average execution time for each query (based on a sample of 10)**

	FedX(first run)	FedX(cached)		TopFed	
Query no	Execution time(msec)	Execution time(msec)	S.E	Execution time(msec)	S.E
1	913	401.2	5.22	341.5*	5.60
2	81619	81170.7	655.93	866.5*	22.08
3	82271	81817.8	653.22	666*	27.12
4	1199	367.6	6.88	262.7*	7.35
5	80423	78723.5	459.43	78691.5	458.70
6	837	416.9	8.38	246.1*	3.56
7	921	399.6	4.41	248.1*	7.20
8	900	89	2.45	72.7*	1.52
9	950.3	76.8	2.16	63.3*	1.89
10	912	63.6	1.99	49.6*	1.02
Average	25094.53	24352.67	180.01	8150.8	53.60

^*^Significant improvement.

We have performed a two-tailed heteroscedastic t-test based on a sample of 10 (each query was run 10 times) to compare the source selection execution time. The source selection execution time and the standard error (S.E) obtained are presented in Table 8. On average, our source selection algorithm only requires 17 msec per query. This is because our N3 specification file is much smaller (only 43 lines) and we have created an in-memory Sesame repository to load and access this file. For the first run, the FedX source selection execution time is much higher. This delay is caused by the query engine sending a SPARQL ASK query for each of the query triple patterns, and for each of the sources. As explained above, FedX needs to issue 102 SPARQL ASK queries to perform source selection for the query in Listing 6 and the data distribution in Figure 3. In order to minimise the number of SPARQL ASK queries, FedX makes use of the cache to store the result of the recent SPARQL ASK request. Every time a query is issued, the engine first looks for a cache hit before issuing the actual SPARQL ASK query. To show the effect of the cache, we have rerun the same query 10 times after the first run and we have noticed a reasonable improvement. For a complete cached entries (100% cache hit), our source selection execution time is still comparable with FedX. It is important to note that all queries that are not specific to a patient (i.e, queries 2, 3, 5, 10), the TopFed source selection time is small (less than 10 msec). The reason is that the tumour number cannot be inferred from these queries and as a result less computation (index lookups) is required in the source selection Algorithm 1.

**Table 8:** **Comparison of source selection average execution time (based on a sampling of 10)**

	FedX(first run)	FedX(cached)		TopFed	
Query no	Execution time(msec)	Execution time(msec)	S.E	Execution time(msec)	S.E
1	530	11.7	0.35	28.1	0.98
2	487	11.4	0.67	5.2	0.57
3	470	11.9	0.78	5	0.42
4	510	12	0.52	23.6	1.57
5	473	9.8	0.65	4.8	0.29
6	371	9.9	0.38	21.7	0.68
7	521	10	0.39	24.4	0.76
8	483	9.5	0.45	29.5	0.86
9	496	9.8	0.39	20.1	0.99
10	456	10.6	0.40	7.4	0.58
Average	479.7	10.66	0.50	16.98	0.77

In Table 7, we compare the execution time of TopFed and FedEx for all of the benchmark queries using a two-tailed heteroscedastic t-test based on a sample of 10. It is important to mention that the query execution time was measured when the first result was retrieved, i.e., we did not iterate over all results. As an overall performance evaluation, the query execution time of TopFed is about one third to that of FedX. Specifically, TopFed significantly outperforms FedX in benchmark queries 2 and 3 related to exon expression and methylation, respectively. These queries select the complete set of results for all of the 25 patients. TopFed is able to infer from the query that the category colour should be pink and green, respectively, and issue the complete query to only the endpoints in the corresponding colour categories. In contrast, FedX is not able to perform such pre-processing, hence issuing the query to all endpoints. As a result, it has to collect results from all of the endpoints in the blue, pink, and green categories when only one of the categories can produce results for each query. As an example of the FedX approach addressing query 2, the triple pattern (?recordNo, tcga:chromosome, ?chromosom) relies on retrieving the results from all of the endpoints in both the blue and green categories, only to return an empty set of results, after making a star join with the triple pattern (?recordNo, tcga:RPKM, ?RPKM). We expect that our approach will generally lead to much faster resolution for queries of this nature, where a large number of triples is retrieved for a specific colour category. This reflects the improvement that TopFed’s engine is able to determine those queries that will return empty sets prior to requesting the data. Although the benchmark query 5 results in a very large set of triples, the execution time for both systems is almost the same. As pointed out above, the reason for this is that the query is too generic and it is impossible to infer the category colour or tumour number.

## Conclusion

In this work, we have published a Linked Data version of TCGA data level 3 (to the best of our knowledge the largest Linked Data dump anywhere) and further linked it to the LOD cloud. This big data resource is designed to be used as infrastructure for biomedical and bioinformatics applications that analyse and query both the file annotations but also the internal content of the patient-derived files of this key reference for molecular biology and epidemiology of cancer.

Anticipating usages that traverse to other related big data resources, we have also generated links to other LOD data dumps such as HGNC, OMIM and Homologene. We believe that this RDFication can greatly help researchers in the biomedical domain as the amount and diversity of data exceeds the ability of local resources to handle its retrieval and parsing. The RDFized data resource can be easily traversed from a modest machine to investigate a variety of measures at each position of the genome, across all types of molecular information, and across all cancer types, without the need to download the files and extract the pieces of information that satisfy the query. In fact, we would argue that this type of analysis will eventually be all but impossible for big data resources like TCGA without RDFication and improved federation schemes such as those described in this paper.

The TCGA data dump (and what we expect will be the genomics datasets in the future) is already too large to be effectively handled by a single server. If the relationships between TCGA and other related resources are taken into account, a smart data distribution framework that distributes the data among multiple SPARQL endpoints, such as the one reported here is, an absolute necessity. This framework, TopFed, is specifically designed as a federated query processing engine that handles a collection of physically distributed RDF data sources. The resulting virtually integrated data resource was observed to enable significantly faster querying and retrieval (one third) than current solutions, such as FedX. The TopFed source selection algorithm achieves this result by considering the metadata about the data distribution with the type of the joins among query triples patterns. The substantial improvements in efficient processing achieved, also in the use of network traffic, suggests that the development of systems designed to process an individual patient clinical data to identify the drugs leading to better outcomes in related cohorts in TCGA-like resources (e.g., ICGC [59]) is now at hand.

One of our future aims is to develop an intelligent system, in which a cancer patient’s genomic data are used as input to suggest effective drugs for treatment while comparing against results from TCGA patients with the same or similar cancer sub-types. In 2009, we contributed to CNViewer [60], a browser based tool that could be used, via oncologists uploading their own patient’s copy number result, to calculate the Euclidean (or other) distance to all other patients with the same tumour type. With TopFed, not only we can calculate these distances using copy number results, but in future work we expect to use aggregation/correlation of molecular results to match and better understand both the biology driving cancer and the most effective treatment for a patient given a set of genetic alterations.

## Availability of supporting data

The TCGA data is available under the original TCGA Data Use Certification Agreement [61] and TopFed source code along with utilities are available under GNU GPL v3 licence at the project home page https://code.google.com/p/topfed/.

## Endnotes

^a^ URLs of SPARQL endpoints hosting five cancer histologies that are shown in Figure 1 can be found at http://tcga.deri.ie/.

^b^ A step-by-step user manual is also available at: http://goo.gl/0oTAKV.

^c^ Available to download from: http://tcga.deri.ie/dumps/.

^d^ See http://www.w3.org/TR/sparql11-query/ for more information on federated queries based on SPARQL 1.1.

^e^ TCGA Data Matrix: https://tcga-data.nci.nih.gov/tcga/dataAccessMatrix.htm.

^f^ Patient barcode format: https://wiki.nci.nih.gov/display/TCGA/TCGA+Barcode.

^g^ Benchmark queries: http://goo.gl/UxUEXk.

^h^ TopFed index: https://topfed.googlecode.com/files/loadDistribution.n3.

## Abbreviations

TCGA: The cancer genome atlas
LOD: Linked open data
S3DB: Simple sloppy semantic database
GBM: Glioblastoma multiforme
RDF: Resource description framework
DHT: Distributed hash tables
GCCs: Genome characterization centres
GSCs: Genome sequencing centres
TSS-to-Tumour: Tissue source site to tumour
S.E: Standard error
ICGC: International cancer genome consortium.
